# In vivo generation of collagen specific Tregs with AAV8 suppresses autoimmune responses and arthritis in DBA1 mice through IL10 production

**DOI:** 10.1038/s41598-021-97739-w

**Published:** 2021-09-14

**Authors:** Matthew Wade, Hugues Fausther-Bovendo, Marc-Antoine De La Vega, Gary Kobinger

**Affiliations:** 1 Department of Microbiology and Immunology, Faculty of Medicine, Laval University, Quebec, Canada; 2 Department of Pathology and Laboratory Medicine, University of Pennsylvania School 27 of Medicine, Philadelphia, PA USA

**Keywords:** Peripheral tolerance, Inflammation, Chronic inflammation

## Abstract

Available therapeutics for autoimmune disorders focused on mitigating symptoms, rather than treating the cause of the disorder. A novel approach using adeno-associated virus (AAV) could restore tolerance to the autoimmune targets and provide a permanent treatment for autoimmune diseases. Here, we evaluated the ability of collagen II T-cell epitopes packaged in adeno-associated virus serotype 8 (AAV-8) vectors to reduce pathogenic cellular and humoral responses against collagen and to mitigate the disease in the collagen-induced arthritis mouse model. The cytokines and immune cells involved in the immune suppression were also investigated. Mice treated with AAV-8 containing collagen II T-cell epitopes demonstrated a significant reduction in the arthritis symptoms, pathogenic collagen specific antibody and T cell responses. The AAV-8 mediated immune suppression was mediated by increased interleukin-10 expression and regulatory T cells expansion. Altogether, this study strengthens the notion that AAV vectors are promising candidates for the treatment of autoimmune diseases.

## Introduction

Rheumatoid arthritis (RA) is a chronic autoimmune disorder with a prevalence in western adult populations of approximately 1%, with a common presentation of small joint inflammation and tissue destruction^1,2^. Despite this relatively high prevalence, RA etiology is not completely understood^2,3^. Due to this incomplete understanding, diagnosis focuses on individual symptom presentation, while treatment aim to minimize chronic disease symptoms such as pain, inflammation, and joint degradation^4–6^. Current treatments include pain management, broadly acting non-steroid anti-inflammatory drugs, corticosteroids, and anti-proliferative medications along with specific biological therapies^7^. These treatments however are only able to stall, and not reverse disease progression. As a result, new avenues of prevention and treatment are being explored using viral vectors for immune modulation.

Minimally immunogenic AAV serotypes such as AAV serotype 8 (AAV8) have been shown to generate robust antigen-specific immunosuppression even when challenged with adenovirus serotype 5 (Ad5) vectors expressing the identical genes^8–10^. These findings led to the testing of AAV8 based therapies in the experimental autoimmune encephalitis (EAE) mice model. In this animal model, administration of AAV8 encoding myelin oligodendrocyte glycoprotein was able to reverse paralysis in mice^8^. In the EAE model, pathogenicity is mediated by auto-reactive T-cells. However, to date there are no reports of successful therapies with AAV vectors in treating what are believed to be antibody mediated autoimmune disorders.

The mechanism of AAV8 induced gene tolerance has been previously explored with the liver being implicated in the ability of AAV8 to induce gene specific tolerance in mouse models^9,11–13^. Within these models, the immune tolerance is thought to be the result of antigen specific peripherally induced CD4^+^CD25^+^FoxP3^+^ T regulatory cells (Tregs)^14^. These Tregs are generated in the liver in part due to the manner in which the secreted antigen is taken up and presented by kuffer cells. This antigen presentation, along with an increased concentration of immunoregulatory cytokines such as interleukin (IL)-10, 13, and transforming growth factor β^10,14–16^ is thought to drive the expansion of hepatic Tregs. These Tregs may only suppress local immune responses in the liver, but may also migrate to exert their regulatory functions at other peripheral sites making AAV8 induced gene tolerance an option for the maintenance or restoration of peripheral tolerance^8,14,17–19^.

The collagen induced arthritis (CIA) model is the most common mouse model of antibody mediated autoimmune arthritis and is an indispensable tool in the study of the pathogenic mechanisms of the disease and testing of therapeutics. This is in large part due to similarities in disorder presentation and tissue destruction, along with its close linkage to MHC alleles^20,21^. Both T and B cell responses to the collagen II (CII) protein are noted in the CIA model and the immunodominant T-cell and B-cell determinants have also been identified^21,22^. Early works in determining CII T-cell epitopes also noted that CII peptide tolerization in susceptible mice can be achieved by adoptive transfer of Tregs modified to present the immunodominant murine epitope of CII (aa259–270) suppressing the disease in recipients^2,21,22^.

Here, we assessed the ability of an AAV8 vectors encoding CII T-cell epitopes to inhibit the symptoms associated with CIA as well as the pathogenic humoral and cellular response against collagen.

## Materials and methods

### Viral construct preparation

Previously characterized CII T-cell epitopes were synthesized in a sequence derived from the native murine collagen II (Col2A1) gene cDNA sequence by Genescript (Piscataway, NJ). The resulting construct was cloned via Gibson assembly (New England Biolabs, Rowley, MA) into either pAAV-MCS or pacAd5 CMV K-N (all from Cell Biolabs, San Diego, CA) to generate the pAAV-MCS: CII or pacAd5 CMV K-N: CII constructs.

### Vector production

HEK293LTV and HEK293Ad cells (Cell Biolabs) were cultivated in Dulbecco’s Modified Eagle Medium (DMEM) (Gibco, Gaithersburg MD) supplemented with 10% Fetal Bovine Serum (Wisent) 1× Penicillin/Streptomycin (Thermofisher, Rockville, MD), 1× Glutamax (Thermofisher). Cells co-transfected with pAAV2/8 (PennVectorCore), pHelper and pAAV-MCSCII or pAAV-MCSGFP (Cell Biolabs) at a molar ratio of 1:1:1 by Calcium Phosphate (Takara, Ann Arbour, MI) according to manufacturer’s directions. Vector purification and titration were performed as previously described^23,24^. Ad5 was produced in HEK293Ad cells. Plasmids pacAd5 CMV K-N: CII and pacAd5 9.2–100 (Cell Biolabs) were co-transfected at a 1:1 molar ratio with Calcium Phosphate (Takara) according to manufacturer’s directions. The virus was released by freeze–thaw and subsequently amplified in HEK293Ad cells and purified as previously described^25^.

### Mice

Female DBA1/J mice (8 weeks of age) were purchased from The Jackson Laboratory (Bar Harbor, MI). Mice were acclimatized for at least a week prior to any experiment. Three mice per group were injected by way of the tail vein with 10^11^ vector genomes (VG) of AAV8 or 10^10^ Ad5 viral particles (VP). Blood and serum samples were collected via cardiac puncture following overdose of isoflurane. In vivo, Treg depletion was performed by intraperitoneal injection of 0.75 mg of PC61 (BioX-cell, West Lebanon, NH) at 2 or 7 days post-AAV administration, while blockage of IL-10 receptor (IL-10R) was achieved with 0.75 mg of 1B1.3A (anti-IL-10R) (BioX-cell) 2 days pre- and post AAV8.CII administration. Induction of CIA and analysis of the progressive inflammatory phenotypic was performed as previously described^26^. All animal protocols were approved by the Institutional Animal Care and Use Committee of Université Laval. All experiments were performed according to the guidelines of the Canadian Council on Animal Care. Throughout the manuscript, the methods of the performed experiments were reported according to the ARRIVE guidelines.

### Liver leukocyte and splenocyte isolation

Liver Leukocytes were isolated by Ficoll. Briefly, the liver homogenate was treated with ACK lysis buffer (Thermofisher) to remove erythrocytes and the cell suspension was subjected to one round of Ficoll (Sigma Aldrich, Nadick, MA) separation. Non-parenchymal cells were recovered and subsequently centrifuged and washed with RPMI (Gibco) supplemented with 10% Fetal Bovine Serum (FBS) (Wisent). Splenocytes were isolated from spleen homogenate and treated with ACK lysis buffer to remove erythrocytes and the resultant cell suspension was suspended in RPMI supplemented with 10% FBS, 1× Penicillin/Streptomycin, 1× Glutamax.

### Interferon-γ enzyme-linked immunosorbent spot assay

The interferon (IFN)-γ enzyme-linked immunosorbent spot (ELISPOT) assay was performed according to the manufacturer’s instructions (BD Biosciences, San Jose, CA)^14^. Briefly, cells were stimulated with 1 μg/ml chicken CII protein (Chondrex, Redmond, WA) for 48 h and spots were visualized with an AEC substrate kit (BD biosciences) and counted with the AID ELISPOT reader system and ISPOT software (version 7, https://www.elispot.com/products/software/)(Autoimmun Diagnostica GMBH, Strabberg, Germany).

### Cell culture and cytokine assays

Plating and culture of liver non-parenchymal cells and splenocytes were performed as previously described^14^. Cells were stimulated with a final concentration of 1 μg/ml chicken CII protein (Chondrex). Interleukin (IL)-10 was detected by an Enzyme-Linked Immunosorbent Assay (ELISA) kit (MabTech) as per the manufacturer’s instructions. IFN-γ was detected by an ELISA kit (R&D Systems, Minneapolis, MN) following the manufacturer’s instructions.

### Proteome profiler cytokine assay

Analysis of cytokines in purified liver non-parenchymal cell culture supernatant was assessed using the Proteome Profiler Cytokine Array Kit, Panel A (R&D Systems) as per manufacturer’s instructions. Spot intensity was assessed using ImageJ (version 1.51; https://imagej.nih.gov/ij/)^27^.

### Flow cytometry

Fresh liver non-parenchymal cells or splenocytes from individual mice were stained with antibodies against B220 (RA3-6B2), CD95 (Jo2), CD3 (SP34-2), CD4 (L200), CD45 (30-F11) were purchased from BD Biosciences (San Jose, CA) and intracellular staining made use of the FoxP3/Transcription factor buffer set (Thermofisher). Data were obtained with a BD Aria II Flow cytometer and were analyzed with the FlowJo Software, version 10.7 (www.flowjo.com). (BD Biosciences).

### Statistics

Statistical analysis was significance assessed by Unpaired T-test or ANOVA using GraphPad Prism version 8.0.0, (GraphPad Software, San Diego, CA). A *p* < 0.05 was considered significant.

## Results

### AAV8.CII injection mitigates the CII specific immune response in DBA1 mice

To date, the ability of AAV to suppress antibody-mediated autoimmunity had not been tested. Here, we sought to evaluate the therapeutic efficacy of AAV8 in the mouse CIA model where pathogenicity is mediated by auto-reactive antibodies against collagen^21^. However, due to the limited packaging size of AAV, the native CII cDNA sequence could not be used. Instead, a targeted cDNA construct was designed to contain all known CII T-cell epitopes. We anticipated that the resulting AAV8.CII would generated regulatory T cells specific to collagen II capable of suppressing both cellular and humoral response toward this epitope.

**Figure 1:**
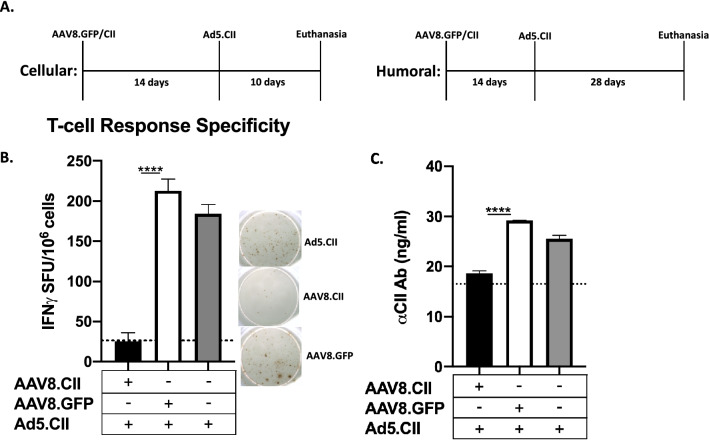
AAV8 mediated tolerance is antigen specific. (**A**) Mice (n = 3/group) were injected intravenously with 10^11^ VG of AAV8.CII or AAV8.GFP. At day 14, mice were injected intravenously with 10^10^ VP Ad5.CII. Control mice were injected with phosphate-buffered saline (PBS). (**B**) 10 days after Ad5.CII injection, Liver non-parenchymal cells were stimulated with CII and IFNγ production was measured by ELISPOT. Spots were analyzed using the ISPOT software (version 7, https://www.elispot.com/products/software/). Background SFU values are indicated by a horizontal dashed line. (**C**) 28 days post Ad5.CII injection Anti-CII IgG levels were measured using ELISA. Background CII reactivity is indicated by a horizontal dashed line. Means ± SEM are illustrated for the different groups.

First, we evaluated the ability of AAV8.CII to prevent collagen specific humoral and cellular responses when challenged with Ad5.CII encoding for the same CII epitopes. Ad5.CII has been shown to trigger immunity to CII mimicking an auto-immune status against this antigen. Mice first received a tail vein injection with 10^11^ VG of AAV8.CII or AAV8.GFP, the later used as a non-specific antigen control. Mice were then challenged 14 days later with 10^10^ Ad5.CII VP (Fig. 1A) as previously described^14^. T cell and antibody responses were measured 10 days and 28 days post Ad5.CII challenge respectively (Fig. 1A). When animals were treated with AAV8.CII prior to Ad5.CII, only background levels of T-cell responses to CII were detected by IFNγ-ELIPOST (~ 25 spot forming units (SFU)/10^6^). This is in contrast to when mice received AAV8.GFP or PBS control prior to Ad5.CII challenges, with no difference of CII specific T cells (200 and 180 SFU/10^6^ cells respectively) being noted in these animals (Fig. 1B). The humoral response against CII demonstrated a similar pattern of reduction in animals receiving AAV8.CII prophylaxis, where they exhibited background levels of anti-CII IgG (19 ng/ml)). Mice receiving AAV8.GFP or PBS prior to Ad5.CII challenge showed no difference in their anti-CII IgG concentration (30 and 28 ng/ml respectively) (Fig. 1C). Taken together these findings indicate that both the cellular and humoral response to CII can be suppressed with AAV8.CII prophylaxis in DBA1 mice. These results also suggest that the AAV8 based immune regulation is transgene specific, as the same AAV8.GFP did not demonstrate a reduction in either T-cell or humoral responses.

### Treg are a critical mediator in AAV8.CII mediated autoimmune suppression

Hepatic Treg have been previously implicated in AAV8-mediated immune suppression^18^. In order to assess their involvement in the suppression of CII immune responses, the frequency of hepatic Tregs was measured by flow cytometry in control and AAV8.CII treated mice. Live Treg were defined by the co-expression of CD45, CD3, CD4, CD25 and Foxp3+, and the absence of staining by a LIVE/DEAD dye (Supplementary Fig. S1). Compared with control mice, a significant increase in Treg average frequency from 6 to 12% of CD4+ T-cells was noted in mice receiving AAV8.CII prophylaxis (Supplementary Fig. S1).

**Figure 2:**
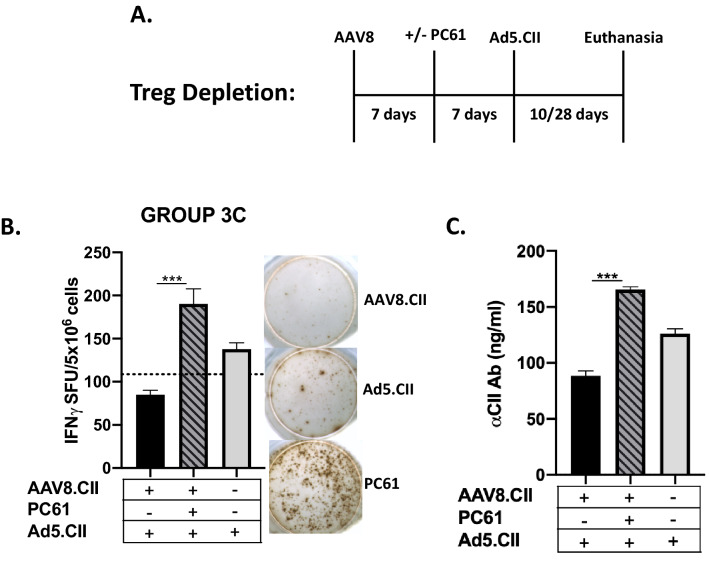
AAV8.CII induced Tregs are crucial for tolerance. (**A**) Mice (n = 3/group) were injected intravenously with 10^11^ VG of AAV8.CII. At Day 7 Mice were injected intraperitoneally with saline or 0.75 mg of PC61 anti-CD25 antibody. 7 days after PC61 injection mice were injected intravenously with 10^10^ VP Ad5.CII. Control mice were injected with phosphate-buffered saline (PBS) alone. (**B**) 10 days after PC61 injection liver non-parenchymal cells were isolated and stimulated with CII and IFNγ production was measured by ELISPOT, with spots detection performed using the ISPOT software (version 7, https://www.elispot.com/products/software/). (**C**) 28 days post PC61 injection, anti-CII IgG levels were measured using ELISA. Background values are indicated by a horizontal dashed line. Means ± SEM are depicted (**B,C**).

Depletion of Tregs was performed prior to AAV8.CII treatment in mice to assess the role of this expanded population on T and B cell responses. For Treg depletion, mice received an intraperitoneal injection of 0.75 mg of PC61, an antibody against CD25, 7 days post AAV8.CII injection (Supplementary Fig. S2). Mice were subsequently injected with Ad5.CII 7 days later (Fig. 2A). Mice only injected with Ad5.CII were used as positive response controls. The frequency of CII-specific T cells was analysed by IFNγ ELISPOT 10 days after the Ad5.CII injection. The magnitude of the humoral response against CII was analysed by CII ELISA 28 days following the Ad5 injection (Fig. 2B,C). Treg depletion in AAV8.CII pre-treated mice led to significant increase in CII-specific T cells as well as CII-specific IgG after Ad5.CII injection (*p* = 0.001 and 0.001 respectively). Indeed, CII-specific T cells increased from 109 SFU/10^6^ (SEM 13.47) in mock treated to 185 SFU/10^6^ cells (SEM 17.27) in depleted mice (Fig. 2B). Similarly, the amount of CII specific IgG nearly doubled from 90 to 150 ng/ml on average between mock treated and Treg depleted mice (Fig. 2C). Of note, an increase in both CII specific cellular and humoral response was also observed in PC61 treated mice even in absence of Ad5 injections (Supplementary Fig. S3).

Overall, a significant decrease in Treg cells was noted in the mice receiving PC61, and this decrease in Tregs was concomitant with an increase in CII-specific cellular and humoral response. Taken together, these data suggest that Tregs are involved in the AAV8.CII mediated suppression of the CII immune response.

### IL-10 is modulated and important to AAV8.CII mediated suppression of CII specific immunity

**Figure 3:**
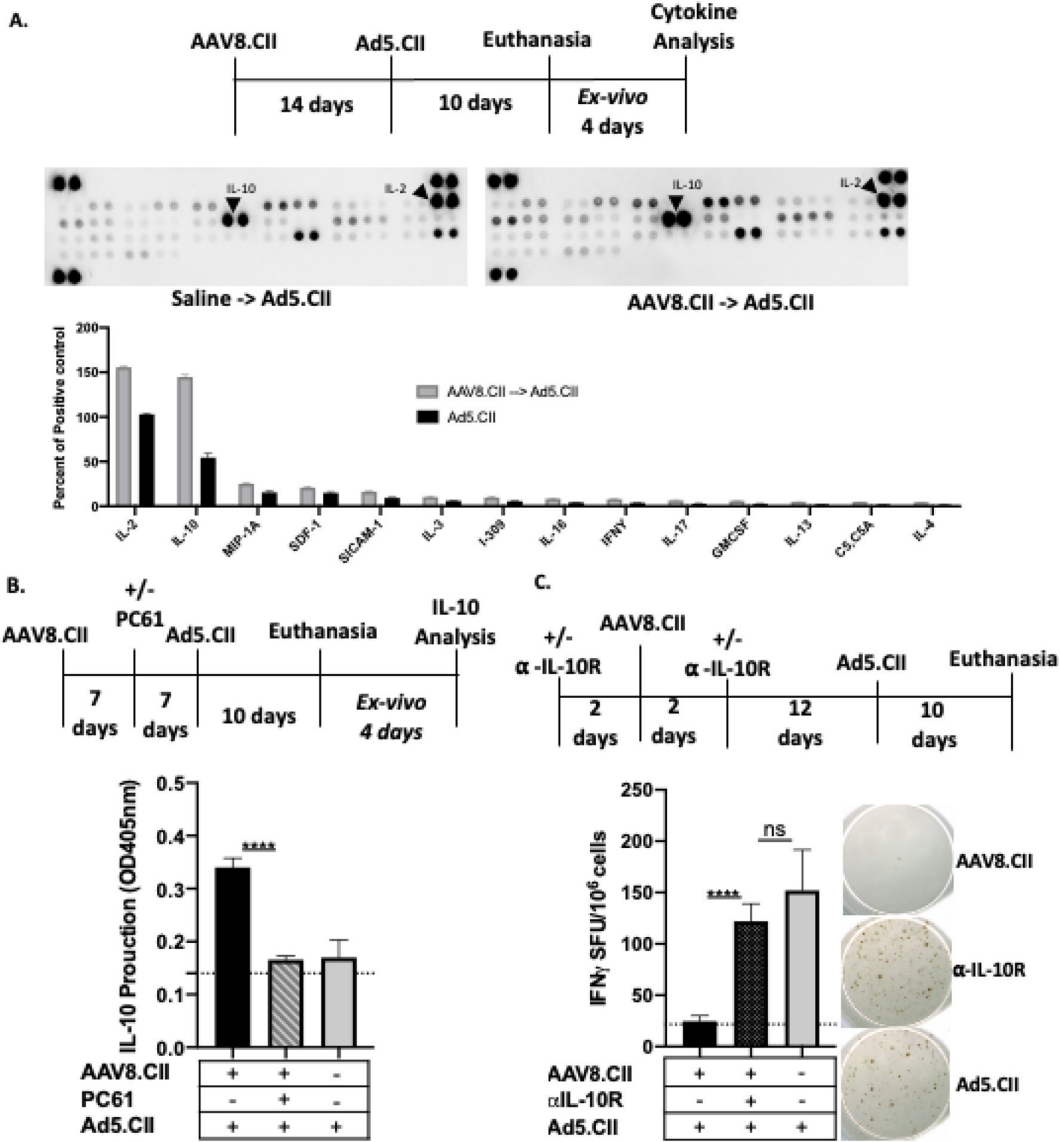
Elevated IL-10 is essential for tolerance to CII. (**A**) Mice (n = 3/group) were injected intravenously with 10^11^ VG of AAV8.CII. At day 14 mice were injected intravenously with 10^10^ VP Ad5.CII. 10 days after Ad5.CII injection liver non-parenchymal cells were cultured with anti-CD3 and anti-CD28 in the presence of CII; 96-h later culture supernatants were collected, pooled and analyzed for cytokine production. Dot blot analysis of cytokine production from cultured liver non-parenchymal cells stimulated with CII. Pixel density was measured with ImageJ software (version 1.51; https://imagej.nih.gov/ij/). (**B**) Quantification of IL-10 production in cell culture supernatants was analyzed by ELISA. Mice were treated as in A except that 0.75 mg of PC61 anti-CD25 antibody was injected in some mice 7 days after AAV8.CII administration (**C**) 10 days after Ad5.CII injection, liver non-parenchymal cells were stimulated with CII and IFNγ production was measured by ELISPOT. Spots were quantified using the ISPOT software (version 7, https://www.elispot.com/products/software/). Mice were treated as in A except that one group of mice which received 0.75 mg of 1B1.3A anti-IL-10R antibody intraperitoneally 2 days prior and 2 days post AAV8.CII injection. (**A–C**) For each group, the mean ± SEM is shown.

To better understand the soluble mediators involved in the AAV8.CII Treg dependent immune modulation. The cytokines profile of CII stimulated liver non-parenchymal cells from control and AAV8.CII treated mice were evaluated by immunoblot. To do so, mice were saline or AAV8.CII treated 14 days prior to Ad5 injection and euthanized 10 days later. Non-parenchymal cells were isolated from liver homogenate by Ficoll and a total of 10^6^ cells were stimulated with 0.1 ug/ml CII for 4 days prior to supernatant collection (Fig. 3A). The expression of 22 cytokines was then profiled using a cytokine proteome profiler. Overall, mice receiving AAV8.CII prophylaxis appears to have slightly elevated cytokine response in comparison to mice receiving only Ad5.CII. However, a more than twofold increase in expression level was only detected for IL-10 (Fig. 3A).

To further explore the origin of IL-10, the above experiment was repeated with the additional of a group in which Tregs were depleted using PC61. The level of IL-10 in the supernatant of CII stimulated hepatic non-parenchymal cells was measured by ELISA. In culture from hepatic non-parenchymal cells originated from AAV8.CII but not Treg depleted or mock treated mice, IL-10 production was elevated with the OD increasing from background levels to an OD of 0.35. In mice that received PC61 post AAV8.CII administration, no increase in IL-10 above background levels was observed following ex-vivo stimulation (Fig. 3B). These results indicate that the detected IL-10 production in the response to CII is tied to Treg cells.

Finally, we sought to evaluate the importance of IL-10 in AAV8.CII mediated inhibition of CII specific cellular response. To do so, mice were treated as in Fig. 3C and CII specific IFNγ production was measured via ELISPOT. A group of mice that received Ad5.CII alone was used as control (Fig. 3C). In mice receiving Ad5.CII alone, approximately 150 SFU/10^6^ CII-specific T cells were detected, while in AAV8.CII prophylactically treated mice, the CII-specific T cells response was present only at background levels (20 SFU/10^6^ cells). Interestingly, AAV8.CII prophylactically treated mice injected with the IL-10R blocking mAb demonstrated CII-specific T cells response at an average of 125 SFU/10^6^ cells. Together these findings suggest that AAV8.CII suppression of CII specific immunity is mediated at least in large part by IL-10, and that this IL-10 is primarily derived from Treg cells in response to CII.

### AAV8 mediated hepatic gene expression mitigates the progression of collagen-induced arthritis

**Figure 4:**
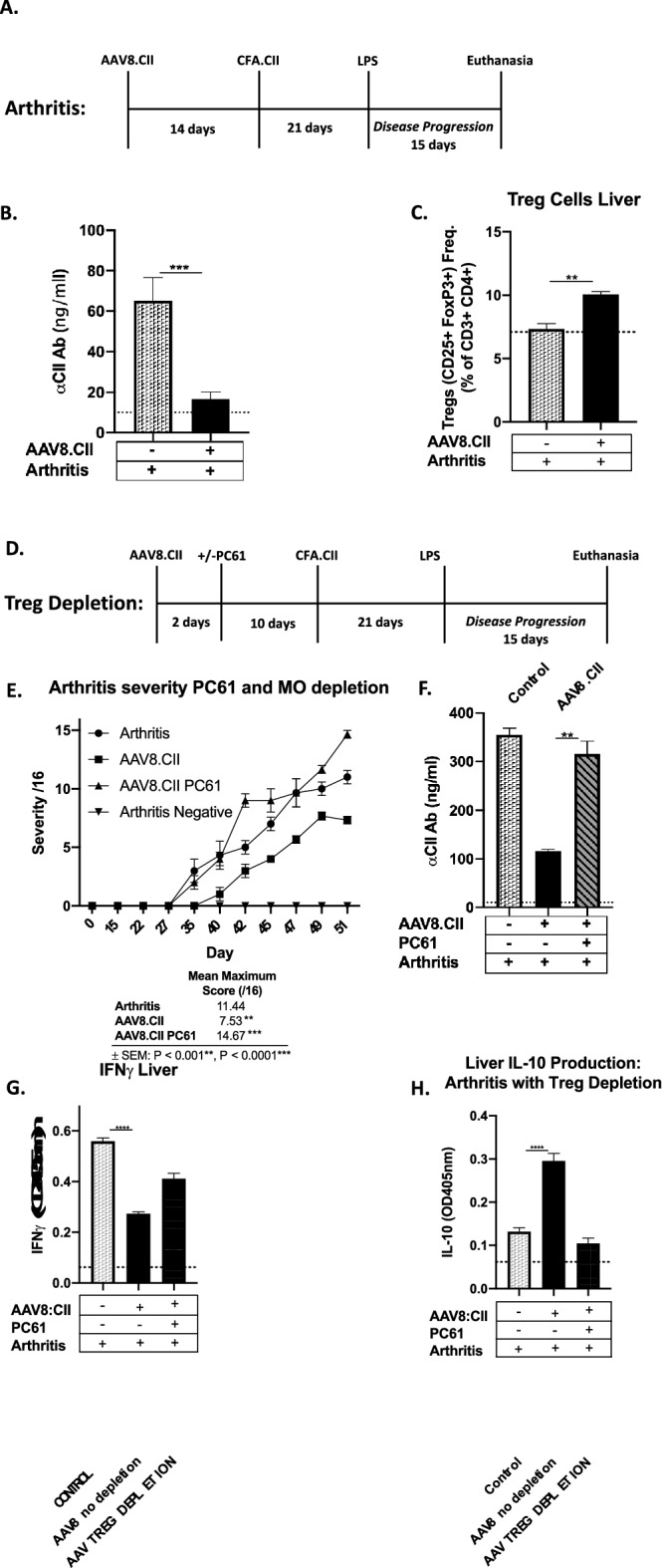
AAV8.CII prophylaxis reduces CIA severity through the induction of Tregs and IL-10 production. (**A–H**) Mice (n = 5/group) were injected intravenously with 10^11^ VG of AAV8.CII. 14 days later they received 100 ug CII emulsified in 100 ug CFA by subcutaneous injection. 21 days later the mice were injected intraperitoneally with 50 ug of LPS suspended in PBS. Peripheral inflammation was followed for the next 15 days and prior to euthanasia. (**A**) Experimental timeline is illustrated (**B**) Serum taken 15 days post LPS injection was assessed for anti-CII IgG by ELISA. (**C**) 15 days post LPS injection, liver non-parenchymal cells were isolated and stained with CD4, CD25 and FoxP3 antibodies run by FACS and the percentage of triple positive cells were compared using FlowJo, version 10.7 (www.flowjo.com). (**D**) AAV8.CII was injected and disease induction performed as per (**A**) with an additional administration of PC61 on day 2. (**E**) Peripheral inflammation was measured in the paws of arthritis mice every 2–3 days. Data presented is the summation of the four paws to a maximum inflammation score of 16 per day. (**F**) Serum taken 15 days post LPS injection was assessed for anti-CII IgG by ELISA. (G&H) IFNγ and IL-10 production in cell supernatant was assessed as in 3.D. by ELISA. (**B,C,F–H**) Means ± SEM are depicted for the various assays.

Based on the above AAV8.CII suppression of CII specific humoral and cellular responses, we explored the possibility of AAV8.CII inhibition on the severity of CIA in DBA1 mice. Mice received either AAV8.CII or saline prophylaxis treatment 14 days before the induction of the disorder and were followed for 15 days after LPS induced disease onset (Fig. 4A). Disease progression was graded based on joint inflammation as previously described^26^, while the IgG response against CII was measured by ELISA post-euthanasia as described above. An approximate 35% reduction in paw inflammation was noted in the mice receiving AAV8.CII prophylaxis (Supplementary Fig. S4). In addition, in AAV8.CII treated mice, there was a significant decrease in anti-CII IgG from 60 ng/ml in mock treated mice to 20 ng/ml in mice receiving AAV8.CII (Fig. 4B). Together, these data suggests that prophylactic administration of AAV8.CII can slow arthritis progression in the CIA model and decrease autoantibody production.

### Both Treg cells and IL-10 are involved in the suppression of arthritis with AAV8.CII prophylaxis

Next, we examine whether Treg and IL-10 were involved in the AAV8.CII-mediated reduction of the arthritis phenotype. First, the frequency of Treg in hepatic non-parenchymal cells was monitored by FACS at the time of euthanasia in CIA induced mice in the presence or absence of AAV8.CII. The frequency of hepatic Tregs increased from 7.3 to 10.1% of CD4 T cells in mice receiving AAV8.CII when compared to saline controls (Fig. 4C). This expansion of Treg cells is consistent with what was observed in the Ad5.CII challenge model where a 5% increase in hepatic Tregs was noted prior to Ad5.CII, and suggests that mechanisms of immunoregulation in the CII specific response are shared between the Ad5.CII challenge, CIA models.

To confirm the suspected role of Treg in AAV8.CII arthritis mitigation, Treg depletion was performed in AAV8.CII treated mice prior to arthritis induction using PC61 (Fig. 4D). Disease progression and level of CII specific IgG was monitored as above. The depletion of Tregs in AAV8.CII treated mice abrogated the reduction in disease progression and resulted in greater peripheral inflammation than arthritis controls (Fig. 4E). The elimination of Tregs also restored the levels of CII specific IgG to the same level as mice with CIA induction alone (Fig. 4F).

Finally, the impact of Treg depletion on IFNγ and IL-10 production in the CIA model was evaluated. To do this, the hepatic non-parenchymal cells from CIA induced mice were isolated with and without Treg depletion and stimulated ex-vivo with CII as described above. Mice that received AAV8.CII prophylaxis had an approximate 50% reduction in the OD of pro-inflammatory IFNγ and a concomitant 2.5-fold increase in IL-10 level when compared to arthritis controls. Treg depletion with PC61 restored the CII specific IFNγ response and eliminated the increased IL-10 production normally observed with AAV8.CII prophylaxis (Fig. 4G,H). This increase in inflammation severity, CII specific antibody, increase in pro-inflammatory cytokine production and decreased immunoregulatory cytokine expression strongly suggests that peripherally induced Tregs are one of the primary mediators of the immunoregulatory response in CIA mice receiving AAV8.CII prophylaxis.

## Discussion

Current RA therapeutics, such as anti-inflammatory, immunosuppressive, and immunomodulating drug regimens, have been used to slow clinical progression of RA with some promising results^5^. However, these treatments, are often non-specific, provide mainly temporary relief, are unable to induce complete disease remission^4,6^. Due to their broad spectrum action, existing treatments suppress the immune system broadly therefore rendering treated individuals susceptible to opportunistic infections and secondary complications^28–31^. Novel countermeasures against RA are therefore needed.

Additional therapeutic candidates against RA are in pre-clinical stages of development. In the mice model of RA, a lentiviral based therapy was able to mitigate disease progression and severity via the induction of antigen specific tolerance to the collagen II peptide^2^. Similarly, administration of Human mesenchymal stem cell grafts, chebulanin, anti-citrullinated peptide antibody, or synthetic compounds such as PEPITEM have all shown promise in mitigating onset or progression of the disease in the collagen induced arthritis mouse model^32–34^.

Unlike biological therapeutics targeting inflammatory cytokines along with the administration of IL-10, AAV-based therapies could provide tolerance targeted to the epitopes in its transgene. In a mouse model of multiple sclerosis, AAV encoding the full myelin oligodendrocyte glycoprotein could mitigate the induction and severity of the disease^8^. This is also supported by the data obtained with AAV8.GFP and the absence of an immunoregulatory phenotype during the Ad5.CII challenge. This suggests that AAV expressing epitopes recognized in autoimmune disorders may be able to specifically suppress pathogenic responses in humans. This specific immunoregulation would eliminate the general immunosuppression seen in existing biological therapies and may not require repeated administration as AAV can stably express antigens in vivo for over 10 years^35^.

It is worth noting that AAVs can only package relatively small transgenes of up to ~ 4 kb and this fact has been a major drawback in its clinical use^36^. Numerous human proteins involved in autoimmunity are indeed larger than 4 kb necessitating a tailored approach to epitope expression. Here we demonstrated that selectively packaging T cell epitopes (amino acids (aa) aa 161–200, aa 245–270 and aa 275–315 of collagen II) from the protein of interest is sufficient to diminish both cellular and humoral responses against the transgene^21,22^. By using epitopes rather than whole proteins, tolerance could theoretically be induced against any antigen, independent of its size.

The crucial role played by central and peripherally induced Tregs in immune regulation has been previously reported in animal models and in human autoimmune diseases^8,9,37^. Lower numbers or functional impairment of Tregs is reported in multiple autoimmune disorders including RA, multiple sclerosis and lupus^8,14,38,39^. The current data also show that Tregs play a central role in regulating CIA with AAV8.CII treatment leading to expanded Treg populations, reduced IFNγ and increased IL-10 in mice^8,12^. This central role of Tregs was further defined with depletion of Tregs removing any benefit in AAV8.CII treated mice and restored tissue damage and IFNγ autoimmune responses against collagen.

The present study shows that the delivery of specific autoimmune targets with AAV8 is able to reduce pathogenic autoimmune responses in mice. Importantly, prophylactic administration of the AAV8.CII vector was able to mitigate the severity of CIA. Due to the speed of disease induction, this model did not allow for evaluation of AAV as a therapeutic agent. Therefore, further exploration in slower acting animal models of autoimmunity must be explored in order to determine AAV therapeutic efficacy in established autoimmune disorders. This work warrants further investigation in order to advance AAV8 based therapeutic of autoimmune syndromes to the clinic.

## Supplementary Information

Supplementary Information.
