# Supplementary material for: In vivo generation of collagen specific Tregs with AAV8 suppresses autoimmune responses and arthritis in DBA1 mice through IL10 production
Source: Sci Rep. 2021 Sep 14;11:18204. doi: 10.1038/s41598-021-97739-w (PMC8440515; doi:10.1038/s41598-021-97739-w)
Supplement: Supplementary file 1 — Supplementary Information. [file 41598_2021_97739_MOESM1_ESM.docx]

**Supplementary figures**


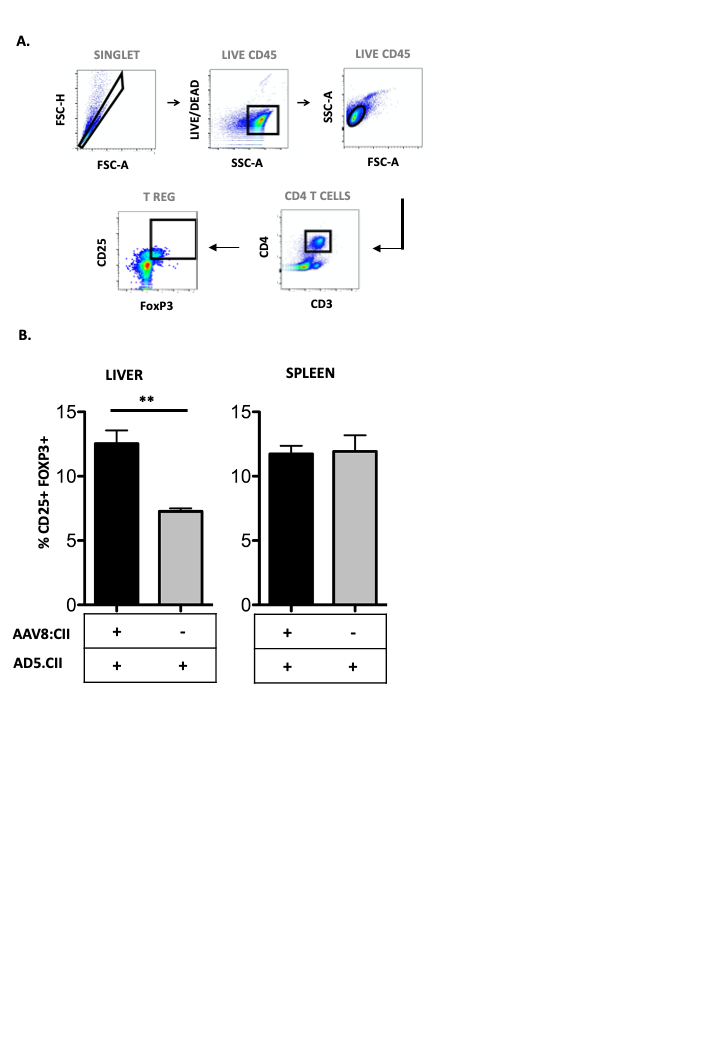


Figure S1: AAV8 treatment increases Treg frequency in the Liver. DBA1/J mice (n=3/group) were injected intravenously with 10^11^ VG of AAV8.CII followed with 10^10^ VP Ad5.CII intravenously 14 days later. Mice were euthanized 10 days thereafter. Cells suspension were obtained from the liver and spleen, then stained with lineage markers and a viability dye. The frequency of Treg was then monitored by FACS.( A.) Gating strategy aimed at quantifying the presence of T reg (Live CD3+ CD4+FoxP3+ CD25+ ). (B.) Hepatic and splenic Treg frequency are indicated in animal treated with or without AAV8-CII. FACS analysis was performed using the FlowJo Software, version 10.7 ([www.flowjo.com](http://www.flowjo.com)).


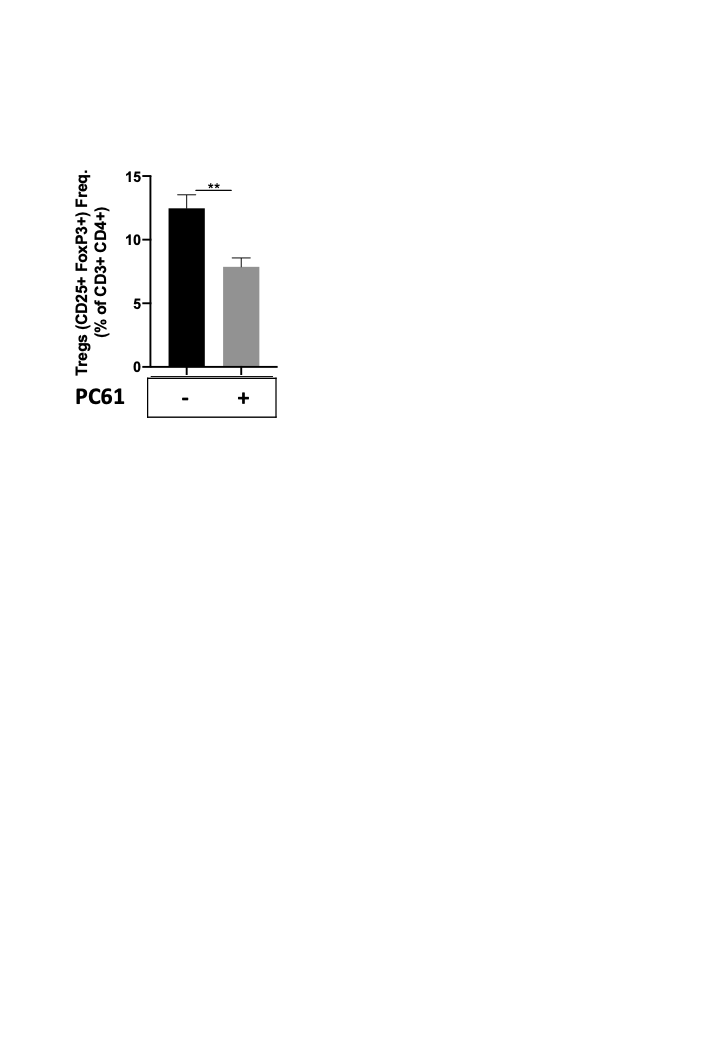


**Figure S2: Treg depletion efficiency following PC61 administration.** Mice (n=3/group) were injected intraperitoneally with 0.75mg of PC61 antibody or an equivalent volume of phosphate buffered saline. 10 days after the injection, splenocytes were isolated and the quantities of CD25+ FoxP3+ Tregs in the population of CD4+ CD3+ T cells were run by FACS and analysed using FlowJo (version 10.7, [www.flowjo.com](http://www.flowjo.com)). Mean Treg frequency +/- SEM are depicted.


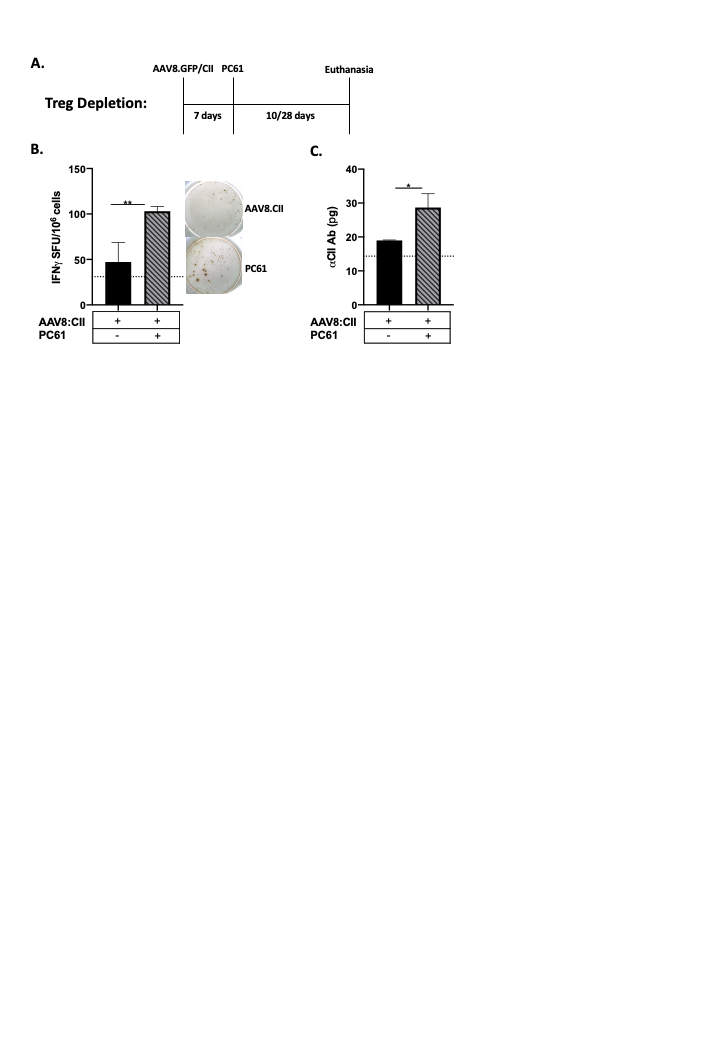


**Figure S3: Treg depletion increased cellular and humoral responses against CII**. (A) Mice (n=3/group) were injected intravenously with 10^11^ VG of AAV8.CII. At Day 7 Mice were injected intraperitoneally with saline or 0.75 mg of PC61 anti-CD25 antibody. (B) 10 days after PC61 injection liver non-parenchymal cells were isolated and stimulated with CII and IFN𝛄 production was measured by ELISPOT. The ISPOT software (version 7, <https://www.elispot.com/products/software/>) was used to analysed spot number (C) 28 days post PC61 injection, anti-CII IgG levels were measured using ELISA. Background values are indicated by a horizontal dashed line. Mean +/- SEM is illustrated for both assays.

**Figure S4: AAV8.CII Prophylaxis reduced severity of arthritis.** Peripheral inflammation was measured in the paws of arthritis mice (n=5/group) every 2-3 days. Data presented is the summation of the four paws to a maximum inflammation score of 16 per day.
